# The Relationship Between Sleep, Cognition and Behavior in Children With Newly-Diagnosed Epilepsy Over 36 Months

**DOI:** 10.3389/fneur.2022.903137

**Published:** 2022-07-26

**Authors:** Temitayo Oyegbile-Chidi, Danielle Harvey, Jordan Eisner, David Dunn, Jana Jones, Anna Byars, Bruce Hermann, Joan Austin

**Affiliations:** ^1^Department of Neurology, School of Medicine, University of California, Davis, Sacramento, CA, United States; ^2^Department of Public Health Sciences, School of Medicine, University of California, Davis, Sacramento, CA, United States; ^3^Department of Psychiatry, School of Medicine, Indiana University, Indianapolis, IN, United States; ^4^Department of Neurology, University of Wisconsin School of Medicine and Public Health, University of Wisconsin-Madison, Madison, WI, United States; ^5^Department of Pediatrics, Cincinnati Children's Hospital at the University of Cincinnati, University of Cincinnati, Cincinnati, OH, United States; ^6^School of Nursing, Indiana University, Indianapolis, IN, United States

**Keywords:** epilepsy, sleep disturbances, pediatric, cognition, behavior, mood, longitudinal, epilepsy comorbidity

## Abstract

**Introduction:**

There is substantial evidence that children with epilepsy experience more sleep, behavior and cognitive challenges than children without epilepsy. However, the literature is limited in describing the relationship between sleep, epilepsy, cognition and behavioral challenges and the interactions amongst these factors over time. This study aims to understand the nature and strength of the relationship between sleep, cognition, mood and behavior in children with new-onset epilepsy as assessed by multiple informants at multiple time periods using multiple different dependent measures.

**Methods:**

332 participants (6–16years) were recruited within 6 weeks of their first recognized seizure. The comparison group was comprised of 266 healthy siblings. Participants underwent sleep evaluation by a parent using the Sleep Behavioral Questionnaire (SBQ), cognitive evaluation using a comprehensive neuropsychological test battery, a behavioral evaluation using the Child Behavior Checklist (CBCL from parents and TRF from teachers) and the Children's Depression Inventory (CDI). These evaluations were completed at baseline (B), at 18 months, and at 36 months.

**Results:**

Compared to siblings, children with new-onset epilepsy had more sleep disturbance (SBQ), higher rates of behavioral problems (CBCL and TRF), lower cognitive testing scores, and higher rates of depression; which persisted over the 36-month study. Sleep significantly correlated with behavioral problems, cognitive scores and depression. When divided into categories based of sleep disturbance scores, 39.7% of children with epilepsy experienced “Persistently Abnormal Sleep”, while 14.8% experienced “Persistently Normal Sleep”. Children with persistently abnormal sleep experienced the highest rates of behavioral problems, depression and cognitive impairment compared to those with persistently normal sleep, regardless of epilepsy syndrome. Younger age of seizure onset, younger age at testing, and lower grade level at baseline were associated with persistently abnormal sleep.

**Conclusions:**

To our knowledge, this is the first demonstration of the nature, strength, reliability, stability and persistence of the relationship between sleep, cognition, and behavioral problems over time in a large cohort of children with newly diagnosed epilepsy, as assessed by multiple informants at different timepoints. The results of this study indicate that children with epilepsy are at a high risk of significant persisting neurobehavioral multimorbidity. Therefore, early screening for these challenges may be essential for optimizing quality of life long-term.

## Introduction

Over the last several decades, it has become increasingly evident that children with epilepsy can exhibit abnormalities in sleep, behavior, and cognition (1, 2). Children with epilepsy experience increased challenges that span across all aspects of life including school performance, behavior, and mood. These challenges do not disappear between seizures and instead permeate throughout the child's life with potential long-term sequela (3). As a consequence, the impact of epilepsy on a child's life is more pervasive than the intermittent clinical seizures may suggest (4).

The interconnection between sleep, behavior, cognition, and epilepsy appears to be significant but poorly understood and the patterns of causality among these factors are uncertain as well (5). On the one hand, sleep disruptions in children with epilepsy may have negative implications on brain function; leading to higher levels of behavioral problems and poorer performance on cognitive testing (6, 7). On the other hand, behavioral problems in children with epilepsy, such as hyperactivity and mood disorder symptoms can adversely affect sleep, causing sleep disruptions, which can manifest as poor attention and worse academic performance at school (8–10). This indicates that the relationship between sleep, behavior and cognition in children with epilepsy is closely intertwined and complex (11).

Critically, while these multiple co-morbidities in children with epilepsy have been well documented, the direct associations between them have yet to be established as they are most often evaluated individually (12–18). In addition, daytime behavioral problems are often provided primarily from parental reports and whether similar relationships would be observed in the reports from the children themselves or from their teachers remain to be determined (19–21). Assessing these co-morbidities concurrently over time across multiple informants would inform on the stability of each distinct co-morbidity and the stability of sleep-behavior-cognition relationships across informants; consequently unveiling the salience and reliability of the relationships. However, the consistency of these relationships has yet to be determined.

In the last few years, we have also begun to note heterogeneity in these abnormalities such that some children with epilepsy exhibit minimal impairment in sleep, behavior and cognition while others show significant impairment in these factors (11, 22). A clearer understanding of the heterogeneity of specific sleep-behavior-cognition patterns in children with epilepsy would be necessary to optimize care and improve quality of life for children with epilepsy.

The existence, stability and reliability of sleep-behavior-cognition relationships over time in children with epilepsy and the clinical consequences of the daytime behavioral and cognitive problems remain current gaps in the literature that we propose to address here. In addition, assessing these relationships in the context of potential heterogeneity is necessary to gain a clear representation of the variable extent of morbidity in these children with epilepsy. The current prospective study examines these relationships in a sample of 332 children with new onset seizures over a 36-month period. The purpose of this study was to characterize the relationships between sleep, cognition, and behavior over time in children with newly-diagnosed epilepsy as assessed by multiple informants (children, parents, teachers,) using multiple assessment tools (CBCL, TRF, CDI and a validated cognitive assessment battery) over 3 distinct time points (baseline, 18 months later, 36 months later). We also attempt to determine patterns in these relationships, assessing the evidence of heterogeneity in this large cohort of children with newly diagnosed epilepsy.

## Methods

### Participants

This study emanated from an investigation of children with new onset seizures, their siblings as controls, and their primary caregivers (23, 24). The core investigation was conducted at Indiana University and Cincinnati Children's Hospital at the University of Cincinnati. A total of 332 children were recruited within 6 weeks of their first recognized seizure (Mean = 35 days). Children were recruited through electroencephalogram (EEG) laboratories, emergency departments, and pediatric neurologists in two large children's hospitals (Indianapolis and Cincinnati) and from practices of private pediatric neurologists in Indianapolis. All children in this sample had recurrent seizures during the duration of the study except for 57 who had only a single seizure during the duration of the study – 20 of them started medications soon after the commencement of the study and 18 demonstrated epileptiform activity on EEG. The sibling control sample was a comparison group of 266 healthy siblings of the children with epilepsy. Only one sibling was recruited per family.

Exclusion criteria for both children with epilepsy and siblings were: a co-morbid chronic physical disorder, intellectual disability (based on either clinic records or parent report), or seizures precipitated by an acute event (e.g., intracranial infection, metabolic derangement, and recent head injury). Children who had had two or more febrile but no afebrile seizures or who were placed on daily antiseizure medication (ASD) after a febrile seizure were also excluded. Parental informed consent and child assent were obtained prior to data collection. Siblings did not have epilepsy and were not on medication that could affect mental status. The study was approved by the institutional review boards at Indiana University and Cincinnati Children's Hospital Medical Center.

Data were first collected within 6 weeks of the first recognized seizure (baseline – B) and in their responses the informants were asked to focus on the time-period 6 months prior to the seizure. Data were collected again 18 months later (M18) and finally, 36 months later (M36). Data were collected using computer-assisted, structured telephone interviews with the primary caregiver, who was the mother with very few exceptions.

#### Demographics

Demographic details are outlined in Table 1. Briefly, a total of 332 children with new-onset seizures aged 6–16 years and 266 sibling controls were included in the analyses at baseline. There were no significant differences between the groups except for a trend toward lower IQ (Table 1) (approximately 3.5 points) in the children with new-onset seizures.

**Table 1:** Demographics table.

**Characteristics**	**Sibling controls**	**Children with epilepsy**
Sample Size	266	332
Age (y)	9.68 (3.7)	9.26 (2.6)
Sex M/F	128/138	163/169
IQ	103.58 (12.9)	100.96 (15.3)~

*No significant differences between sibling controls and children with epilepsy. Children with epilepsy had a trend toward a lower IQ compared to sibling controls. Data presented as mean (SD). ~p <0.1*.

For children with epilepsy, the attrition rate over the first 18 months of the investigation was 10% (*N* = 296) and another 5% over the second 18 months (*N* = 279, Table 2). The majority of children with new onset seizures were on medications at some point during the 36 months of this study. The five most frequently prescribed medications were valproic acid, oxcarbazepine, carbamezapine, phenytoin, and lamotrigine. Other less commonly prescribed medications included felbatol, levetiracetam, phenobarbital, ethosuximide, topiramate, zonisamide, and gabapentin.

**Table 2:** Data of children with epilepsy over the three timepoints (B, M18, M36) presented as Mean (SD), except for cognition.

	**Baseline (*N* = 332)**	**M18 (*N* = 296)**	**M36 (*N* = 279)**
SLEEP	50.4 (12.5)	47.3 (10.8)	44.5 (9.7)
CBCL			
- Internalizing problems	55.55 (11.3)	51.59 (11.3)	50.63 (10.7)
- Externalizing problems	52.34 (10.8)	51.51 (10.5)	49.60 (10.8)
- Total problems	55.28 (11.5)	52.31 (11.7)	50.51 (11.4)
TRF			
- Internalizing problems	54.39 (10.2)	53.20 (10.0)	53.34 (10.1)
- Externalizing problems	53.00 (10.3)	51.57 (9.6)	51.62 (9.0)
- Total problems	54.90 (11.0)	53.82 (10.2)	53.46 (9.9)
CDI	8.48 (7.0)	7.66 (6.7)	6.39 (5.7)
COGNITION			
- Language	−0.054	−0.053	0.003
- Executive function	−0.089	0.147	0.355
- Verbal memory/learning	−0.056	−0.013	0.060
- Processing speed	−0.026	−0.097	−0.092

*Cognition presented as mean factor scores*.

### Instruments

#### Sleep Evaluation

The Sleep Behavior Questionnaire (SBQ) was completed by the parent to characterize the child's sleep problems during the prior 6 months (B), 18 months later (M18) and 36 months later (M36). The SBQ has 35 items describing sleep habits and behaviors that are rated using five-point scales of 1 (*never*), 2 (*just a few times*), 3 (*sometimes*), 4 (*quite often*), 5 (*always*). Parents were specifically instructed to exclude any behaviors that might have been actual seizure activity or any unusual sleep behaviors that occurred immediately prior to, during, or after a seizure episode. The reliability and validity of the SBQ as well as norms based on behavior and age have been established in the past (5, 19). This study focused on the summary scores for bedtime difficulties, parent-child interactions, sleep fragmentation, parasomnia, and daytime drowsiness. The specific scales comprised in the summary scores are listed in the Supplementary Material. The final score varies between 26 and 130. The higher the score, the greater the number of sleep problems, which consequently indicates worse sleep disturbance overall.

#### Cognitive Evaluation

All children and sibling controls completed a comprehensive neuropsychological test battery that included standardized clinical measures of intelligence, language, immediate and delayed verbal and visual memory, executive functions, speeded fine motor dexterity, and academic achievement at baseline, M18 and M36. The specific administered tests included were:

Clinical Evaluation of Language Fundamentals, 3^rd^ Edition (CELF-3) (25, 26) – assesses semantics, morphology, syntax, and pragmatics to get a complete picture of the child's language skills.

Comprehensive Test of Phonological Processing (CTOPP) (27, 28) – assesses phonological processing abilities as an indirect measure of reading fluency.

Conners' Continuous Performance Test, 2^nd^ Edition, (CPT-II) (29, 30) – assesses cognitive, behavioral, and emotional problems focusing on ADHD and related co-morbid disorders from the perspective of the parent, teacher and child.

Kaufman Brief Intelligence Test (K-BIT) (31, 32) – assesses verbal and nonverbal intelligence.

Coding and Symbol Search Subtests of the Wechsler Intelligence Scale for Children, 3^rd^ Edition (WISC-III) (33, 34) – assesses processing speed, visual-perceptual and decision-making speed.

Wide Range Assessment of Memory and Learning (WRAML) Design Copy (35, 36) – assesses immediate memory, delayed recall and recognition.

Wisconsin Card Sorting Test (WCST) (37–39) – assesses executive function by measuring preservation and abstract thinking, strategic planning, organized searching, ability to utilize environmental feedback to shift cognitive sets, direct behavior toward achieving a goal and ability to modulate impulsive responding.

Testing was administered by psychometrists who were trained, observed, and certified on the test battery and its scoring by a pediatric neuropsychologist (39).

Each test was administered according to standardized procedures, and scores were converted to age-corrected standardized scores using the best available national norms for all tests except WRAML Design Copy, which was designed by this study's research group and for which no norms are available. Factor analysis of this neuropsychological test data (40, 41) revealed four underlying factors: (1) Language, (2) Processing Speed, (3) Executive Function/attention/construction (EF), and (4) Verbal Memory and Learning (40). The Language factor consisted of measures of verbal concept formation, phonological awareness, and phonological memory. The Processing Speed factor consisted of measures assessing psychomotor speed and rapid naming. The Executive Function (EF) factor consisted of measures assessing sustained attention, problem solving, and visual-construction. The Verbal Memory and Learning factor consisted of measures of rote verbal learning and story recall (40). Higher factor scores indicate better neuropsychological performance.

#### Behavioral Evaluation

Three instruments were used to assess emotional and behavioral concerns - Child Behavior Checklist/4-18 (CBCL from parents & TRF from teachers) and Children's Depression Inventory (CDI-1) (42–44). The CBCL was completed by a caregiver/parent to measure each child's and sibling's behavior problems during the past 6 months, at M18 and at M36. Details of this instrument are provided elsewhere (43). Briefly, the CBCL has 118 items describing behaviors that are rated using 3-point scales of 0 (*not true*), 1 (*somewhat or sometimes true*), and 2 (*very true or often true*) (43). For further information in regards to validity and reliability of the CBCL, see https://aseba.org/reliability-validity-information/. Three summary scores from the CBCL were used including t-scores for Total Behavior Problems, Total Internalizing Problems, and Total Externalizing Problems, all normed for age and biological sex. For the children with seizures, parents were specifically instructed to exclude any behaviors that might have represented actual seizure activity or any behaviors that occurred immediately prior to, or after, a seizure. The TRF was completed by each child's teacher (45) based on the child's current behavior at B, M18 and M36 following the child's first seizure to assess baseline neuropsychological functioning and temperament. Details of this instrument are also provided elsewhere (45–48). The TRF was completed by one teacher only (primary teacher) per time period, who usually was a different primary teacher at each time period. Like the CBCL, each item was rated on a 3-point scale and scores were computed for the three broadband scales: Total Behavior Problems, Internalizing Problems, and Externalizing Problems.

Both the CBCL and TRF have been used extensively in children with epilepsy (49–53). Many past studies have relied primarily upon parents to rate their child's behavior problems (23, 54, 55). Making use of both the CBCL and TRF provides insight into informant consistency and lends credence to the reliability of the behavior problems of the child as seen in multiple different settings (school and home primarily).

The CDI-1 is a self-report questionnaire for children and adolescents designed to identify symptoms of depression in developmental age (56). The children with seizures completed this measure at B, M18 and M36.

### Statistical Analysis

All data obtained were collated and analyzed using the Statistical Package for Social Sciences (SPSS) software (Version 27.0, IBM, Chicago IL). Mixed effects analysis was employed to compare cognition and behavior scores in children with epilepsy and siblings. This statistical approach was used to address potential confounds of using sibling controls instead of typically developing healthy controls without an epilepsy sibling. After sleep pattern groupings were established (details below), univariate analysis of variance was used to assess sleep pattern groups amongst children with seizures to evaluate differences in baseline epilepsy characteristics, cognition and behavior. Least significant difference (LSD) was used for *post hoc* testing. All analyses controlled for age, biological sex and number of medications.

## Results

### Characteristics of Sleep, Behavior and Cognition Over 36 Months

A summary of the sleep, behavior and cognition data at baseline, 18 months later (M18) and 36 months later (M36) is detailed in Tables 2, 3. Compared to published controls (Average Sleep Score = 38.2) (19, 40), children with seizures show a higher level of sleep problems that remains consistently elevated over the 36-month study period. Compared to sibling controls, behavior problems as reported by both parents and teachers were more problematic (higher) in children with seizures, while cognitive testing scores were lower in children with seizures. Both behavioral problems and cognitive impairment remained consistently elevated over the 36-month period compared to sibling controls (Table 3). In addition, children with seizures show higher depression scores consistently over 36 months as well.

**Table 3:** Mixed effects modeling indicates that children with seizures have significantly more behavioral problems (t-scores) and perform poorer across all cognitive domains (factor analysis scores) compared to sibling controls.

	**Baseline**		**M18**		**M36**	
	**Sibling controls**	**Children with seizures**	**Sibling controls**	**Children with seizures**	**Sibling controls**	**Children with seizures**
CBCL						
- Internalizing problems	49.93 (11.1)	55.55 (11.3)**	47.36 (10.4)	51.59 (11.3)**	47.09 (10.2)	50.63 (10.7)**
- Externalizing problems	50.56 (11.9)	52.34 (10.8)~	49.32 (11.2)	51.51 (10.5)**	48.70 (10.7)	49.60 (10.8)
- Total problems	50.08 (12.2)	55.28 (11.5)**	47.53 (11.7)	52.31 (11.7)**	47.01 (11.5)	50.51 (11.4)**
Cognition						
- Language	0.125	−0.054*	0.310	−0.053**	0.323	0.003**
- Executive function	0.169	−0.089**	0.375	0.147*	0.490	0.355~
- Verbal memory/learning	0.119	−0.056**	0.259	−0.013**	0.280	0.060**
- Processing speed	0.189	−0.026*	0.263	−0.097**	0.325	−0.092**

CBCL data presented as means (SD). *p < 0.05, **p < 0.005.

#### Interrelationship Between Sleep, Behavior and Cognition Over 36 Months

Sleep and behavior problems as well as sleep and cognitive problems in children with seizures were significantly interrelated and the relationship remained significant over 3 years (see Table 4). Using both CBCL and TRF summary scores, parental and teacher assessments of the child's behavior were associated with sleep and remained consistent across timepoints. Of note, the correlations between Sleep and CBCL appear stronger/higher than Sleep and TRF, possibly due to different raters each year amongst the teachers. Sleep and cognition as well as sleep and depression scores were also correlated over the 3 timepoints.

**Table 4:** Correlational findings.

	**Baseline**	**18 months later**	**36 months later**
Sleep & CBCL			
Internalizing	0.469**	0.477**	0.457**
Externalizing	0.419**	0.419**	0.402**
Total problems	0.503**	0.484**	0.506**
Sleep & TRF			
Internalizing	0.256**	0.076	0.032
Externalizing	0.160**	0.156*	0.156*
Total problems	0.250**	0.171**	0.140*
Sleep & CDI	0.156*	0.219**	0.174**
Sleep & cognition			
Language	−0.149*	−0.217**	−0.040
Executive function	−0.241**	−0.266**	−0.156*
Verbal memory/learning	−0.154*	−0.212**	−0.108
Processing speed	−0.115*	−0.164*	−0.113

Sleep findings correlate significantly with behavioral problems (CBCL > TRF), depression scores, and cognition scores. Data presented as Pearson's correlations, *p < 0.05, **p < 0.005.

### Sleep Patterns Over Time

The above interrelationships suggest that a deeper evaluation may provide further insight on a clinical categorization of sleep problems and its correlates, offering more clinically meaningful evidence. As such, we investigated for different sleep patterns amongst children with seizures. In prior studies, average sleep problems in epilepsy scored at 54 and average sleep problems in healthy controls is scored at 38.2, with standard deviation of 6 (19, 40). Based on this, Normal sleep was set at ≤ 44 and Abnormal sleep at ≥45. In this large cohort of children with seizures who were monitored at 3 timepoints over a 36-month period, we determined the following groupings:

Persistently Normal Sleep defined as normal ( ≤ 44) at B, M18, and M36

Worsening Sleep defined as normal at B but worsened at a time point over the next 3 years

Improving Sleep defined as abnormal at B but improved at a time point over the next 3 years

Persistently Abnormal Sleep defined as abnormal sleep (≥45) at B, M18, and M36

In this large cohort, 40% of children with seizures fell into the persistently abnormal group and 85.2% of children with seizures had abnormal sleep at any timepoint during the 36 months (Table 5). Within the persistently abnormal sleep group, bedtime difficulties and parent-child interactions tended to be the most affected category of sleep problems. The persistently normal sleep group showed significantly lower sleep problems scores compared to the persistently abnormal sleep group (F3, 297) = 87.6, *p* < 0.001).

**Table 5:** Breakdown of sleep patterns over 36 months using sleep scores ranging from 26–130.

	**Persistently normal (14.8%)**	**Worsening sleep (22.6%)**	**Improving sleep (22.9%)**	**Persistently abnormal (39.7%)**
Bedtime difficulties	41.99	45.79	56.78	67.40
Parent-child interactions	35.47	39.06	58.58	69.17
Fragmented sleep	40.05	44.25	56.09	59.02
Parasomnias	37.50	39.93	51.53	61.83
Daytime drowsiness	51.04	52.20	59.65	59.65

*Almost 40% of children with seizures show persistently abnormal sleep, with bedtime difficulties and parent-child interactions being the categories with the highest level of sleep problems*.

### Sleep Patterns and Seizure Category

Seizures in this large cohort of children fell into six syndrome categories - generalized tonic clonic seizures (GTCs), absence seizures, simple partial seizures with and without secondary generalization, complex partial seizures with and without secondary generalization, atonic seizures-myoclonic seizures-akinetic seizures, and unknown. There were no significant differences by syndrome [F_(3, 299)_ = 1.253, *p* = 0.291, Table 6]. There were also no significant differences when assessed by generalized seizure syndromes versus focal seizure syndromes [F_(3, 299)_ = 0.433, *p* = 0.729, Table 7]. In addition, there were no significant differences by one versus more than one type of seizure semiology [F_(3, 299)_ = 0.188, *p* = 0.904, Table 7].

**Table 6:** Sleep patterns broken down by Seizure Syndromes.

	**Total**	**Normal**	**Worsening sleep**	**Improving sleep**	**Persistently abnormal**
GTC	24%	23.3%	26.9%	26.1%	24.6%
Absence	12.6%	18.6%	11.9%	17.4%	10.5%
Simple Partial	15.2%	14.0%	13.4%	14.5%	15.8%
Complex Partial	41.5%	39.5%	35.8%	36.2%	43.0%
Akinetic/atonic/myoclonic	1.2%	0%	1.5%	1.4%	0.9%
Unknown	5.6%	4.7%	10.4%	4.3%	5.3%

*CPS, Complex Partial Seizures; GTC, Generalized Tonic Clonic Seizures; There were no significant differences by seizure syndrome*.

**Table 7:** Baseline clinical characteristics by sleep pattern groups.

	**Total**	**Normal**	**Worsening sleep**	**Improving sleep**	**Persistently abnormal**	***P-*value**
Age	9.26 (2.55)	9.19 (2.56)	10.10 (2.78)	9.28 (2.30)	8.77 (2.57)**	0.015
Sex	51.9% F	52.3% F	50.7% F	52.9% F	47.5% F	0.665
Education	3.77 (2.45)	3.79 (2.08)	4.52 (2.80)	3.82 (2.26)	3.29 (2.39)*	0.02
Age of onset	9.56 (2.53)	9.51 (2.20)	10.35 (2.82)	9.57 (2.32)	9.1 (2.51)*	0.025
Number of seizures	44.69 (176.08)	30.26 (154.66)	38.95 (179.16)	63.49 (216.94)	42.8 (155.64)	0.79
Most common seizure syndrome	41.5% CPS	39.5% CPS	35.8% CPS	36.2% CPS	43.0% CPS	0.291
% Generalized seizures	38.6%	31.8%	38.8%	37.1%	37.3%	0.729
# Seizure types (% ≥2 seizures	8.7%	6.8%	10.4%	8.6%	10.2%	0.904
Race	78.8% W	86.4% W	86% W	84% W	80% W	0.460
Income	$40–50k	$50–60k	$40–50k	$40–50k	$40–50k	0.434
Education of caregiver	13.82 (2.25)	13.74 (2.25)	13.54 (2.10)	14.13years (2.23)	13.82 (2.36)	0.543

Age, education level, and age of onset were significantly different amongst the groups, such that younger age, younger age of onset and lower grade level were associated with persistently abnormal sleep. CPS, Complex Partial Seizures. *p <0.05, **p <0.005, ~p <0.1.

### Relationship Between Sleep Patterns on Behavior and Cognition as Assessed by Multiple Informants

#### CBCL

**Figure 1:**
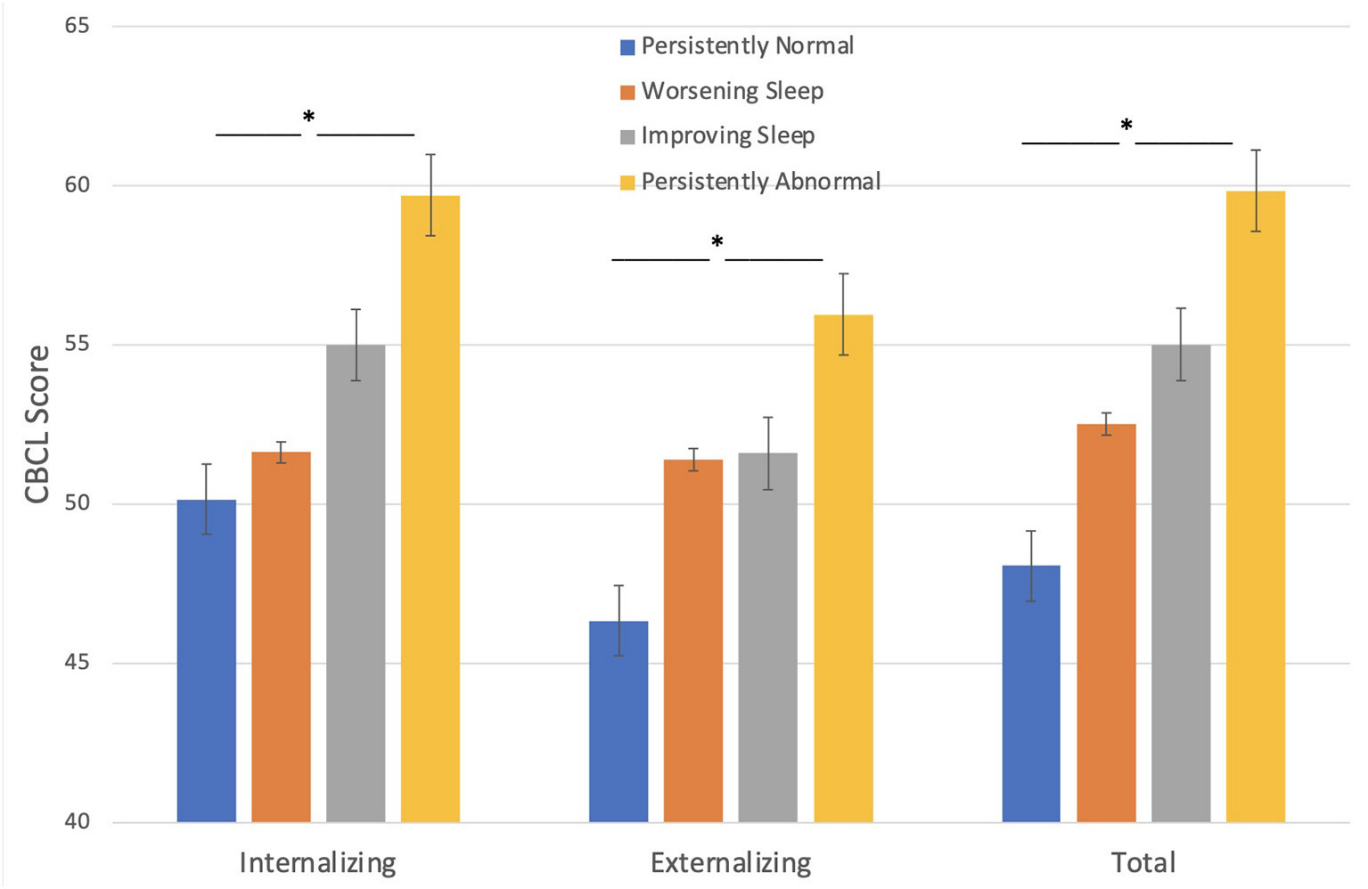
Sleep groups and Behavior problems (CBCL). Those with persistently abnormal sleep exhibit a higher level of behavioral problems compared to those with persistently normal sleep. *p < 0.05.

Behavioral problems at baseline, as assessed by parents, were significantly higher in children with seizures who fell into the persistently abnormal sleep group compared to those with persistently normal sleep across the three timepoints (Figure 1). Sleep groups significantly differ amongst CBCL internalizing problems [F_(3, 265)_ = 11.59, *p* < 0.001], externalizing problems [F_(3, 265)_ = 8.40, *p* < 0.001], and total problems [F_(3, 265)_ = 13.92, *p* < 0.001] such that those with a persistently abnormal sleep pattern show a higher level of behavioral problems compared to those with a persistently normal sleep pattern.

#### TRF

**Figure 2:**
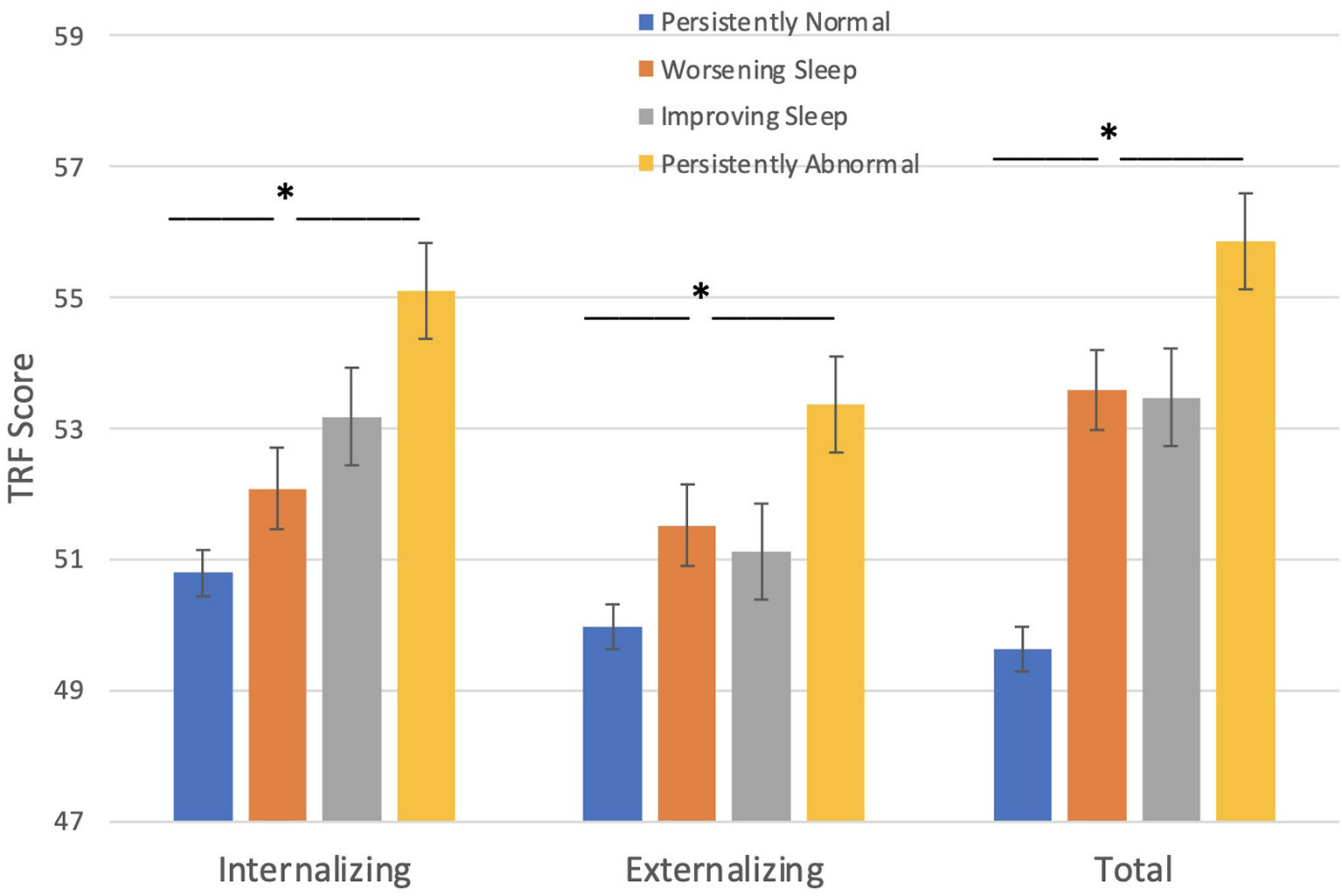
Sleep groups and Behavior problems (TRF). Those with persistent abnormal sleep exhibit a higher level of behavioral problems compared to those with persistently normal sleep. *p < 0.05.

Behavioral problems, as assessed by teachers, were significantly higher in children with seizures who fell into the persistently abnormal sleep pattern compared to those with a persistently normal sleep pattern across the three timepoints (Figure 2). Sleep groups significantly differ amongst TRF internalizing problems [F_(3, 223)_ = 3.25, *p* = 0.023], externalizing problems [F_(3, 223)_ = 4.22, *p* = 0.006], and total problems [F_(3, 223)_ = 7.37, *p* < 0.001] such that those with a persistently abnormal sleep pattern show a higher level of behavioral problems compared to those with a persistently normal sleep pattern.

#### CDI

Depression scores were significantly higher in children with seizures who fell into the persistently abnormal sleep pattern compared to those with a persistently normal sleep pattern across the three timepoints [F_(3, 203)_ = 4.50, *p* 0.004].

#### Cognition

**Figure 3:**
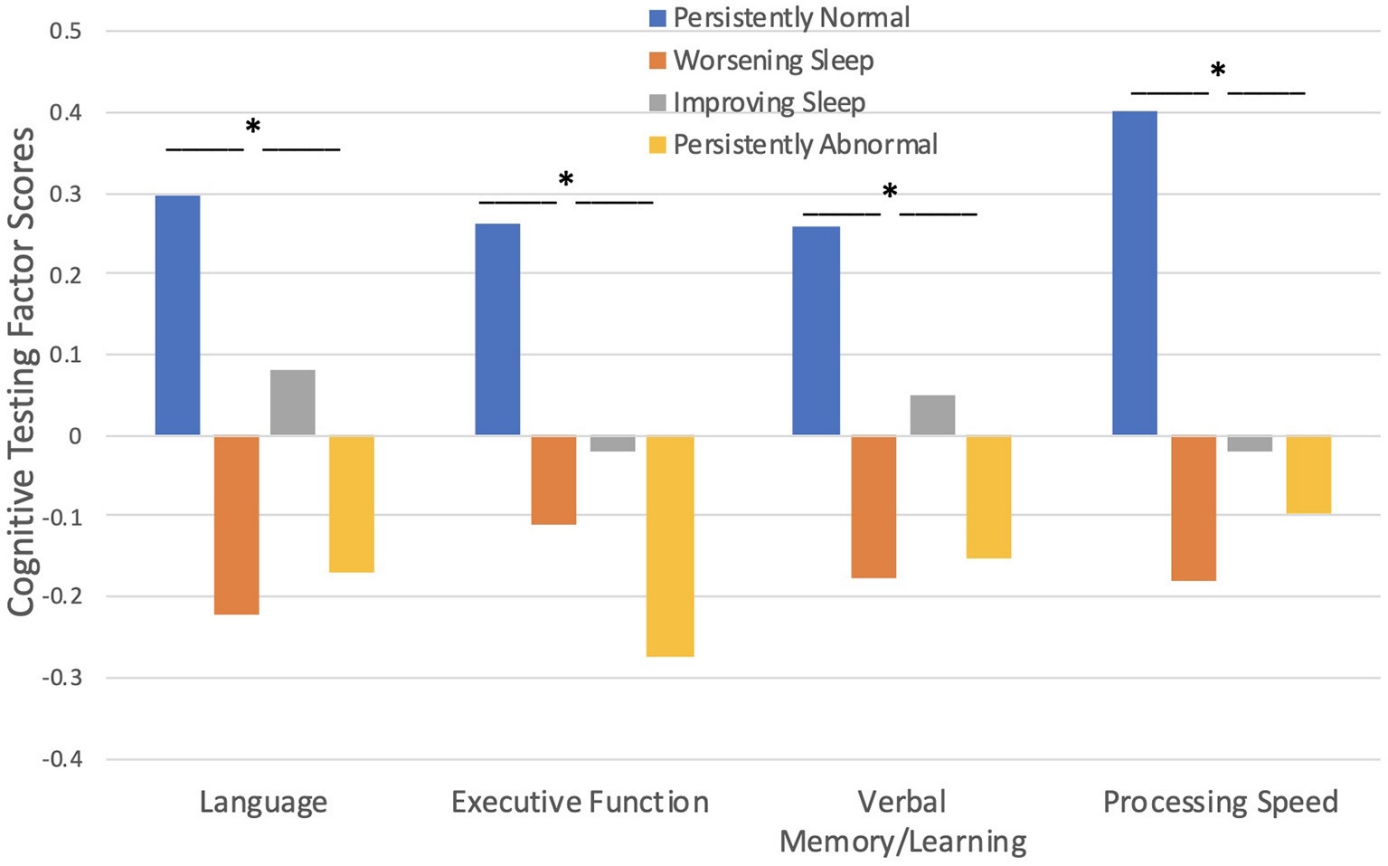
Sleep pattern groups and Cognitive Testing. Those with persistently abnormal sleep exhibited poorer performance on cognitive testing compared to those with persistently normal sleep. *p < 0.05.

Cognitive testing scores were significantly worse in children with seizures who fell into the persistently abnormal sleep pattern compared to those with a persistently normal sleep pattern across the three timepoints (Figure 3). Sleep pattern groups significantly differ in performance on language [F_(3, 261)_ = 3.95, *p* = 0.009], executive function [F_(3, 261)_ = 4.36, *p* = 0.005], verbal memory/learning [F_(3, 261)_ = 3.20, *p* = 0.024], and processing speed [F_(3, 261)_ = 3.92, *p* = 0.009]; such that those with a persistently abnormal sleep pattern show a higher level of cognitive impairment compared to those with a persistently normal sleep pattern.

### Baseline Clinical Characteristics of Sleep Pattern Groups

Results of univariate analyses, illustrated in Table 7, indicate that age of testing [F_(3, 293)_ = 2.964, *p* = 0.015], education [F_(3, 295)_ = 3.113, *p* = 0.02], and age of onset [F_(3, 293)_ = 2.867, *p* = 0.025] are significantly different between persistently normal sleep and persistently abnormal sleep pattern groups. Specifically, younger age of onset, younger total age, and lower grade level at the baseline timepoint was associated with persistently abnormal sleep patterns.

## Discussion

The main objective of this study was to prospectively assess the presence, extent and persistence of neurobehavioral multimorbidity of epilepsy. Specifically, we assessed the relationship between sleep, cognitive impairment and behavioral problems in children with newly diagnosed seizures using multiple informants (parents, teachers, participants), multiple distinct timepoints, and multiple outcome measures of behavior, sleep, and cognition. In addition, we used sleep patterns over a 36-month period to determine trends within cognitive and behavioral problems amongst these children.

Our results indicate that: (1) Children with newly diagnosed epilepsy exhibit a complex matrix of emotional, cognitive, behavioral and sleep disturbances at the outset of the disorder, and even slightly prior to the diagnosis, as our baseline data were based on the 6 months prior to epilepsy diagnosis. (2) There is a robust, reliable, and persistent interrelationship between sleep, cognition and behavior, which was consistently observed regardless of informant (parents, teachers, children) across four different dependent measures demonstrating remarkable stability in the observed relationships. (3) These relationships are persistent and stable at specific timepoints over the 36-month observation period, suggesting a strong reliable signal. (4) These multiple morbidities – sleep, cognition, behavior problems – were present in all epilepsy syndrome groups. (5) Finally, the results point to the substantial heterogeneity in the multimorbidity that is evident in children with epilepsy–affected by clinically significant sleep, behavior, and cognitive issues.

To further reinforce the concept of multimorbidity in newly diagnosed epilepsy, our unique study evaluated the consistency of sleep patterns over time, using a prospective longitudinal approach. We found that children whose sleep disturbance remained constant over the 36 months also showed consistency in their behavioral and cognitive problems. As such, children with consistently low levels of sleep disturbance had the lowest baseline levels of cognitive impairment, depression, internalizing, externalizing, and total behavior problems; while children with consistently high levels of sleep disturbance had the highest baseline levels of cognitive impairment, depression, internalizing, externalizing, and total behavior problems. This pattern was noted in all epilepsy syndromes tested. It is also important to note the reliability of these sleep-cognition-behavior relationship findings as sleep predicted cognitive and behavioral assessment as noted by multiple informants – children, parents and a different primary teacher each year for 3 years. When comparing the CBCL vs. TRF relationship to sleep disturbance, the relationship appears stronger and more consistent for parental reports compared to teacher reports. It is important to note that teachers changed yearly and this change may contribute to some variability in the correlational relationships over the 3-year period. In spite of this, total behavior problems appear to be most highly correlated with sleep amongst teacher assessments over a 3-year period.

In the last couple decades, there has been an emerging interest in and attention toward the intriguing new discoveries involving the links between cognition, sleep and psychopathology in epilepsy (3, 14, 17, 18, 39, 57–63). To our knowledge, this is the first demonstration of the nature, strength, reliability, stability, and persistence of the interrelationship between sleep, cognitive, and behavioral problems over a long period of time in a large cohort of children with newly diagnosed epilepsy, as assessed by multiple informants at different timepoints. At this point, causality cannot be inferred, however, it is clear that these multi-morbidity patterns are substantial. Most studies tend to investigate these comorbidities individually (15–18, 59–64), however, we show here that investigating these factors simultaneously can provide insight into the overall burden as well as the interrelationships that persist over time in a large cohort of children with seizures.

These interrelationships are of clinical consequence as significant sleep disturbance can affect daytime function – behavior and cognition, which may ultimately adversely affect school performance. For clinical practitioners, it is clear that children with seizures need to be evaluated further to determine if they have sleep, cognitive, emotional, and/or behavioral problems (57, 65). Evaluating and treating sleep and/or emotional/behavioral problems may significantly improve seizure trajectory and academic performance. However, this study takes the indication even further by suggesting that children with specific sleep/behavior patterns may be at higher risk compared to others. In the clinical setting, an ability to determine which sleep and behavioral patterns are at the highest risk is certainly an important consideration for future research. A focus on high-risk children with epilepsy can increase the chances of successful sleep/behavioral interventions early in the course of the disorder and perhaps lead to improved school performance and quality of life overtime (64, 66).

It is important to note that anti-seizure medications could have played a role in our findings as several anti-seizure medications can affect sleep, behavior, and cognition adversely. However, our baseline data and findings are a reflection of sleep and behavior over the 6 months prior to the first seizure and cognition prior to or concomitant with starting anti-seizure medications. This indicates that there is evidence of sleep, behavior and cognitive problems independent of anti-seizure medications, which is consistent with prior literature that cognition and behavior may be abnormal prior to the diagnosis of epilepsy (3, 15). The findings from our large cohort data showed no significant differences amongst epilepsy syndromes, which is consistent with some prior literature (3, 8, 22, 58) but varies from other prior literature (16, 62, 63). In spite of this, the existence of cognitive, behavior and sleep problems is indeed a consistent finding in the literature amongst all epilepsy syndromes, which corroborates our findings. The inferences from our study are also limited as our controls were based on sibling data and published controls. Furthermore, some of the findings were based on subjective data (using well-validated surveys and self-evaluations). More advanced evaluations with polysomnogram and computational sleep and wake electroencephalogram (EEG) analysis would be warranted in future studies to further understand the interrelationships of these multi-morbidities with more objective measures (18).

Future research would ideally determine predictive patterns amongst these multi-morbidities so as to optimize treatment interventions sooner than later. Notably, our study showed that those with consistently high sleep disturbance, high behavioral problems and poorer cognitive performance tended to have a younger age of onset, which could potentially be predictive. More sophisticated precision statistical analyses such as latent trajectory analysis may lead to better predictive phenotyping patterns which would enhance our understanding of the interrelationships of sleep, behavior, cognition in epilepsy and inform on patterns of causality.

## Data Availability Statement

The raw data supporting the conclusions of this article will be made available by the authors, without undue reservation.

## Ethics Statement

The studies involving human participants were reviewed and approved by Indiana University IRB Cincinnati Children's Hospital IRB. Written informed consent to participate in this study was provided by the participants' legal guardian/next of kin.

## Author Contributions

TO-C, BH, and JJ conceptualized and designed the study, drafted the initial manuscript, and reviewed and revised the manuscript. JA, DD, and AB designed and conceptualized the larger original study cohort, designed the data collection instruments, collected data within each of their study sites, carried out the initial analyses of the larger cohort, and reviewed and revised the current manuscript. DH coordinated and supervised the data analysis, conceptualized statistical methods, and critically reviewed the manuscript for important statistical content. JE assisted with statistical analysis, the initial manuscript draft, manuscript formatting, and the bibliography. All authors approved the final manuscript as submitted and agree to be accountable for all aspects of the work.

## Funding

This research was supported by the National Institute of Neurologic Disorders and Stroke (NS22416, J. Austin, P.I.). The project described was also supported by the National Center for Advancing Translational Sciences, National Institutes of Health, through grant number UL1 TR001860 and linked award KL2 TR001859. The content is solely the responsibility of the authors and does not necessarily represent the official views of the NIH.

## Conflict of Interest

The authors declare that the research was conducted in the absence of any commercial or financial relationships that could be construed as a potential conflict of interest.

## Publisher's Note

All claims expressed in this article are solely those of the authors and do not necessarily represent those of their affiliated organizations, or those of the publisher, the editors and the reviewers. Any product that may be evaluated in this article, or claim that may be made by its manufacturer, is not guaranteed or endorsed by the publisher.
