# Supplementary material for: The Relationship Between Sleep, Cognition and Behavior in Children With Newly-Diagnosed Epilepsy Over 36 Months
Source: Front Neurol. 2022 Jul 26;13:903137. doi: 10.3389/fneur.2022.903137 (PMC9360804; doi:10.3389/fneur.2022.903137)
Supplement: Supplementary file 1 [file Table_1.DOCX]

|  | Persistently Normal | Worsening Sleep | Improving Sleep | Persistently Abnormal |
| --- | --- | --- | --- | --- |
| Behavior |  |  |  |  |
| CBCL Int | 50.16 | 51.63 | 55.0 | 59.71** |
| CBCL Ext | 46.34 | 51.4 | 51.6 | 55.95** |
| CBCL Total | 48.06 | 52.52 | 55.02 | 59.85** |
| TRF Int | 50.79 | 52.08 | 53.18 | 55.1** |
| TRF Ext | 49.97 | 51.52 | 51.12 | 53.37** |
| TRF Total | 49.63 | 53.59 | 53.47 | 55.86** |
| CDI | 4.85 | 8.78 | 10.34 | 8.61** |
| Cognition |  |  |  |  |
| Language | .295 | -.222 | .081 | -.170** |
| Executive Function | .263 | -.111 | -.021 | -.275** |
| Verbal Memory/Learning | .258 | -.175 | .051 | -.154* |
| Processing Speed | .401 | -.180 | -.020 | -.098** |

**Supplemental Table 2.** Breakdown of behavior and cognition by sleep pattern groups. Those with persistent abnormal sleep consistently exhibited higher levels of behavioral-emotional problems and poorer performance on cognitive testing compared to those with persistently normal sleep *p<0.05, **p<0.005, ~p<0.1
